# Pediatric osteoporosis

**DOI:** 10.1186/1546-0096-12-S1-I27

**Published:** 2014-09-17

**Authors:** Rolando Cimaz

**Affiliations:** 1 Pediatric Rheumatology, AOU Meyer, Firenze, Italy

## 

Pediatric Osteoporosis (PO) is rarely due to bone primary genetic diseases, more frequently a consequence of chronic illness and/or medical treatments. Considering dual X-ray absorptiometry (DXA) the gold standard, the exact relationship between Bone Mineral Density (BMD) and risk of fracture in children is unknown. Furthermore, BMD measurements in children are affected by body size, since the bone density value produced is an areal bone density (aBMD), failing to provide a true volumetric BMD. Diagnosis should therefore be made on the basis of a low BMD in addition to the presence of a clinically significant fracture history. Newer diagnostic techniques such as bone ultrasound and pQCT seem very promising. Peak bone mass is a major determinant of bone mass later in life, as a consequence it is critical to maximize during childhood and adolescence the potential of reaching its optimal value through management of factors contributing to bone loss. Secondary forms of low bone mass include systemic inflammatory diseases which affect bone metabolism due to proinflammatory cytokines and glucocorticoid use. Failure to develop adequate bone mineralization is common in JIA. Methotrexate has been shown to be toxic for bone, but this effect was not confirmed in studies that evaluated its *in vivo* effect on bone when used for the treatment of JIA. TNF-inhibitors have also been shown *not* to interfer with bone density, likely because they are very effective in decreasing the underlying disease activity. Regarding other paediatric rheumatic diseases, many data have shown that also patients with juvenile SLE are prone to suffer from decreased BMD. Similar findings have been reported for dermatomyositis, in which according to the mechanostat theory muscle disease has an additional negative effect on bone strength. Since the cause of bone loss is frequently multifactorial and the exact pathogenic mechanism of PO has not been clearly established in many conditions, it is difficult to proceed with rational treatments and prevention. The armamentarium of drugs to treat bone fragility in children is limited and most have never been established as safe or effective in randomized controlled trials. Furthermore, while the guidelines for the treatment of osteoporosis in adults are widely accepted, the paucity of data for children and adolescents with PO makes it harder to set clear guidelines for the pediatric population. If there is evidence of vitamin D deficiency and/or poor dietary calcium intake it is appropriate to replace such deficits, but routine calcium and vitamin D supplementation is not recommended. Physical activity in childhood is one of the most powerful preventive strategies in the fight against osteoporosis, especially during the peripubertal years; physical activities shown to have the greatest osteogenic effects on the growing skeleton are those characterized by a considerable loading magnitude applied at a rapid rate. Finally, bisphosphonates are effectively used as principal treatment of bone fragility in some genetic forms, such as osteogenesis imperfecta. The same beneficial effect has been shown in children and adolescents with connective tissue diseases. However, their use still raises some concern for possible unknown long-term side effects and for their theoretical teratogenic potential. Therefore the routine use of bisphosphonates in childhood is still not recommended.

## Disclosure of interest

None declared.

